# Effect of empathy trait on attention to various facial expressions: evidence from N170 and late positive potential (LPP)

**DOI:** 10.1186/1880-6805-33-18

**Published:** 2014-06-30

**Authors:** Damee Choi, Takayuki Nishimura, Midori Motoi, Yuka Egashira, Riko Matsumoto, Shigeki Watanuki

**Affiliations:** 1 Department of Kansei Science, Kyushu University, 4-9-1, Shiobaru, Minami-ku, Fukuoka 815-8540, Japan; 2 Department of Public Health, Nagasaki University Graduate School of Biomedical Sciences, 1-12-4 Sakamoto, Nagasaki, Japan; 3 Faculty of Design, Kyushu University, 4-9-1, Shiobaru, Minami-ku, Fukuoka 815-8540, Japan

**Keywords:** Empathy, Late positive potential, N170, Event-related potential, Attention, Face

## Abstract

**Background:**

The present study sought to clarify the relationship between empathy trait and attention responses to happy, angry, surprised, afraid, and sad facial expressions. As indices of attention, we recorded event-related potentials (ERP) and focused on N170 and late positive potential (LPP) components.

**Methods:**

Twenty-two participants (12 males, 10 females) discriminated facial expressions (happy, angry, surprised, afraid, and sad) from emotionally neutral faces under an oddball paradigm. The empathy trait of participants was measured using the Interpersonal Reactivity Index (IRI, J Pers Soc Psychol 44:113–126, 1983).

**Results:**

Participants with higher IRI scores showed: 1) more negative amplitude of N170 (140 to 200 ms) in the right posterior temporal area elicited by happy, angry, surprised, and afraid faces; 2) more positive amplitude of early LPP (300 to 600 ms) in the parietal area elicited in response to angry and afraid faces; and 3) more positive amplitude of late LPP (600 to 800 ms) in the frontal area elicited in response to happy, angry, surprised, afraid, and sad faces, compared to participants with lower IRI scores.

**Conclusions:**

These results suggest that individuals with high empathy pay attention to various facial expressions more than those with low empathy, from very-early stage (reflected in N170) to late-stage (reflected in LPP) processing of faces.

## Background

Humans are considered social animals, as they have greater and more extensive social cognitive abilities than many other species [1]. To achieve amicable social interactions, it is important for humans to pay attention to the faces of other humans and to discriminate facial expressions accurately. However, attention response to faces is thought to differ depending on characteristics of individuals such as personality, sex, and age.

Given that the face provides such important cues to understanding the emotions and ideas of others, attention response to faces is thought to be deeply related to empathy. Empathy means ‘the ability to imagine oneself in another’s place and understand the other’s feelings, desires, ideas, and actions’ (*Encyclopaedia Britannica*, 1999). Previous neuroscience studies have suggested that facial expressions play an important role in empathic responses [2-4]. For example, in a functional magnetic resonance imaging (fMRI) study by Carr *et al*. [2], imitation and observation of facial expressions activated largely overlapping brain areas (for example, the insula), suggesting that empathy is related to action representation such as imitation of the facial expressions of others. Thus, one factor causing individual differences in attention response to faces is thought to be the empathy trait of individuals. Indeed, some neuroscience studies [5-8] have reported relationships between empathy trait and brain activities evoked by watching faces.

Our previous event-related potentials (ERP) study [5] revealed that empathy trait correlates positively with late positive potential (LPP) elicited while discriminating between happy and angry faces. LPP is a positive potential showing at approximately 200 ms after stimulus onset in the centro-parietal area [9,10] and reflects the motivational significance of stimuli [9,11-13]. Given that the LPP is more positive in response to pleasant or unpleasant stimuli than to neutral stimuli [9,13,14], increased positivity of the LPP thus appears related to increased attention to stimuli [13-17]. That previous study [5] thus suggests that people with high empathy pay more attention than those with low empathy trait when discriminating between happy and angry faces.

Another ERP study by Soria Bauser *et al*. [6] reported that the higher empathy trait participants had, the more negative were the N170 components elicited in response to angry faces. N170 is called a face-selective component, as the negative peak is shown in the posterior temporal areas around 170 ms after face onset [18-22]. N170 is also known to be more negative when faces are attended [21] and to show age-related increases in the right posterior temporal area [22]. The findings of Soria Bauser *et al*. [6] thus imply that a reliable relationship exists between empathy trait and attention response to angry faces, not only in the late stage (300 to 800 ms after stimulus onset) [5], but also in the early stage (170 ms after stimulus onset) of attention.

Both of the two previous studies by Choi and Watanuki [5] and Soria Bauser *et al*. [6] measured the empathy trait of participants using the Interpersonal Reactivity Index (IRI) [23]. The IRI is a questionnaire that assesses the empathy trait using four subscales: perspective taking (scale to represent attempts to take the perspectives of others); fantasy (scale to measure the tendency to be immersed in fiction such as drama and novels); empathic concern (scale related to the tendency to feel compassion for others); and personal distress (scale to measure the discomfort generated in response to observing others in negative or emergency situations) [23]. The IRI has been one of the most widely used indices of empathy trait in other neuroscience studies [7,24-26].

Since Ekman and Friesen [27] investigated the universality of facial expressions of emotion, basic facial expressions have generally been thought to comprise the following six expressions: happiness; anger; surprise; fear; sadness; and disgust. However, very few studies have investigated the relationship between empathy trait and attention to those various facial expressions. As mentioned above, empathy trait in previous studies was correlated with the brain activity elicited by discriminating happy and angry faces [5] or discriminating happy, angry, and neutral faces [6]. In addition, an fMRI study by Jabbi *et al*. [7] found that the empathy trait (as measured by IRI) of participants correlated positively with activation of the anterior insula and adjacent frontal operculum elicited in response to food-related pleased and disgusted facial expressions.

To the best of our knowledge, only one study [8] examined the relationship between empathy trait and attention to more than four expressions, using neurotypical adult participants as subjects. In an fMRI study by Chakrabarti *et al*. [8], the empathy trait of participants was measured using the Empathy Quotient (EQ) [28], with participants observing short movie clips of happy, angry, sad and disgusted faces. The results showed that, across all facial expressions, empathy trait correlated positively with activation of the inferior frontal gyrus and ventral premotor cortex [8]. However, differences were also seen in brain areas which correlated with empathy trait depending on the facial expressions viewed (for example, for happy faces, EQ correlated with ventral striatal response; for angry faces, EQ correlated with precuneal and lateral prefrontal cortical response), suggesting different evolutionary functions of each emotion [8].

However, it is necessary to use ERP to clarify how early empathy trait starts to affect the attention processing of various facial expressions, since ERP provides higher temporal resolution than fMRI. The high temporal resolution of ERP is thought to enable us to clarify whether empathy trait is related to the very early stage (reflected in N170) and late stage (reflected in LPP) of attention to facial expressions.

Moreover, which aspect of empathy is correlated with those facial expressions is still unclear, because the EQ questionnaire [28] used by Chakrabarti *et al*. [8] lacks the subscales reflecting various aspects of empathy included in the IRI. Empathy is a multi-dimensional concept [23,29,30] and has three facets: sharing of experience (which is sharing another’s state); mentalizing (which is considering and understanding another’s state); and prosocial concern (which is expressing motivation to help another) [30]. The IRI subscales are thought to reflect those three facets of empathy. In other words, personal distress and fantasy scales of IRI appear to reflect the sharing of experience facet of empathy, whereas perspective taking scale of IRI appears to reflect the mentalizing facet of empathy. Empathic concern scale of IRI appears to reflect the prosocial concern facet of empathy. Examination of relationships between empathy and attention response to faces using the IRI is thus warranted.

We thus aimed to investigate the relationships between the IRI and ERP responses to five facial expressions (happy, angry, surprised, afraid, and sad), to extend knowledge of the relationship between empathy trait and attention to face. Participants discriminated those five facial expressions from emotionally neutral faces under an oddball paradigm. As indices of attention, the N170 and LPP components of ERP were examined. We predicted that individuals with high empathy trait would pay more attention to all facial expressions (happy, angry, surprised, afraid, and sad) and may thus show a more negative N170 and a more positive LPP compared to individuals with low empathy.

## Methods

### Participants

Twenty-two Japanese university or graduate school students participated in the study (12 men, 10 women; age range, 21 to 28 years; all right-handed). Participants had normal or corrected-to-normal vision and were not using prescription medications. They filled out the Japanese version [31] of the IRI [23] using responses on a scale of 1 (‘does not describe me well’) to 4 (‘describes me very well’). Written informed consent was obtained from all participants prior to participation. All study protocols were approved by the ethics committee in the Department of Design at Kyushu University, Japan.

### Stimuli and procedures

Images of 12 adult humans (6 men, 6 women) showing six types of facial expression (neutral, happy, angry, surprised, afraid, and sad) were taken from the Karolinska Directed Emotional Faces [32] for a total of 72 images. All images were edited to 300 × 400 pixels and presented in the centre of a black screen (17-inch monitor, 1,024 × 768 resolution).

Five blocks of oddball tasks were conducted during ERP recording. In each block, target stimuli were happy, angry, surprised, afraid, or sad faces, while non-target stimuli were emotionally neutral faces in all blocks. Participants were instructed to press a key with the right hand as soon as they saw the target. Each block consisted of 96 trials, during which the target was presented 25% of the time (24 trials). After a cross shape was presented for 500 ms, a target or non-target image was presented for 800 ms (interstimulus interval, 1,000 ms). Targets were never presented on two consecutive trials.

After oddball tasks, participants assessed the valence and arousal of images based on a visual analog scale (VAS) (for valence, 0 cm indicated ‘very pleasant’, the middle part of the scale indicated ‘neutral’, and 10 cm indicated ‘very unpleasant’; for arousal, 0 cm indicated ‘very aroused, the middle part of the scale indicated ‘neutral’, and 10 cm indicated ‘very relaxed’). We scored 0 cm as -10 points and 10 cm as 10 points.

### ERP measurements and analysis

Electroencephalography (EEG) was recorded at the Fz (medial frontal), Cz (medial central), Pz (medial parietal), T5 (left posterior temporal), and T6 (right posterior temporal) sites based on the International 10 to 20 system [33] with averaged ears as reference using a Polymate AP1532 system (TEAC, Tokyo, Japan). Electrooculography (EOG) was recorded to detect blinking with electrodes above and below the right eye. All electrode impedances were below 10 kΩ.

EEG signals were digitized at a sampling rate of 500 Hz and amplified (band pass, 1 to 30 Hz) using the EMSE Suite (Source Signal Imaging, San Diego, CA, USA). We excluded trials containing artifacts > 50 μV and trials during which the subject did not show any response. Target stimulus presentation of -200 to 800 ms was averaged (baseline: stimulus presentation of -200 to 0 ms) for each facial expression (happy, angry, surprised, afraid, and sad). The mean number of trials was 20.6 (standard deviation (SD) = 2.3) for happy faces, 20.0 (SD = 3.6) for angry faces, 21.3 (SD = 3.2) for surprised faces, 20.2 (SD = 3.4) for afraid faces, and 20.1 (SD = 2.9) for sad faces.

N170 was calculated as mean amplitude within 140 to 200 ms at the T5 and T6 sites. LPP was calculated as mean amplitude within 300 to 600 ms (for early LPP) and 600 to 800 ms (for late LPP) at the Fz, Cz, and Pz sites.

### Statistical analysis

For ERP responses, we conducted repeated-measures analysis of variance (ANOVA) with Emotion (happy, angry, surprised, afraid, and sad) and Site (N170: T5 and T6; LPP: Fz, Cz, and Pz) as within-subject factors. We then correlated IRI score with N170 at T6, early LPP at Pz, and late LPP at Fz (Pearson’s correlation coefficient) (for details, refer to Results).

For behavioral data (response accuracies, reaction times, and subjective ratings), we conducted repeated-measures ANOVA with Emotion as a within-subject factor and then correlated IRI score with behavioral data (Pearson’s correlation coefficient).

Statistical significance was accepted at the 5% level (*P* < 0.05) (SPSS, Chicago, IL, USA). The Greenhouse-Geisser correction was applied where sphericity was violated. We analyzed male and female data together, since no significant sex differences in IRI score were apparent (independent *t*-test, equal variances assumed; total IRI score, *t* = -1.35; Perspective taking, *t* = -0.63; Fantasy, *t* = -0.95; Empathic concern, *t* = -1.39; Personal distress, *t* = -0.92, all df = 20, *P* > 0.05).

## Results

### Empathy trait

Table 1 shows the IRI scores of participants.

**Table 1:** Empathy trait (Interpersonal Reactivity Index (IRI) score)

	**Range**	**Mean (SD)**
Total score	54 to 96	79.0 (10.5)
Perspective taking	13 to 27	20.5 (3.9)
Fantasy	11 to 28	19.6 (4.5)
Empathic concern	14 to 25	20.1 (2.8)
Personal distress	13 to 25	18.8 (3.4)

n = 22.

### N170

**Figure 1:**
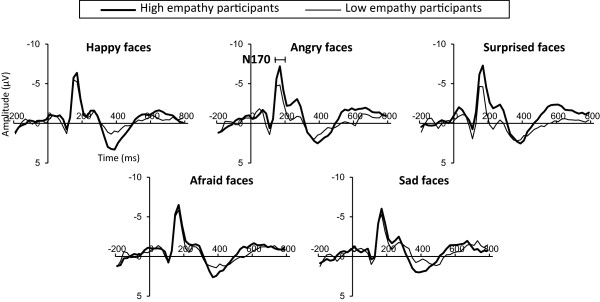
**Grand-averaged event-related potential (ERP) waveforms elicited at T6.** High empathy participants (thick line, n = 12, range of total IRI scores: 80 to 96) and low empathy participants (thin line, n = 10, range of total IRI scores: 54 to 78) were selected by total Interpersonal Reactivity Index (IRI) score and labeled only for illustrative purposes.

**Figure 2:**
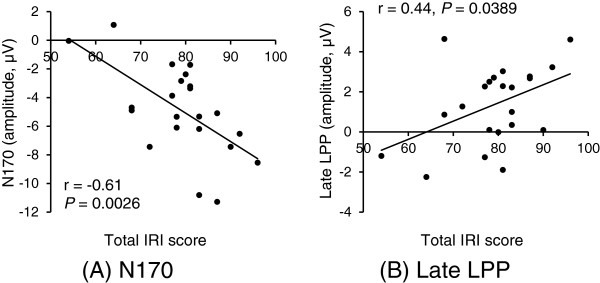
**Correlations between empathy trait (IRI score) and event-related potential (ERP) responses to surprised faces.** Total IRI score correlated negatively with **(A)** N170 (*P* = 0.0026) and positively with **(B)** late positive potential (LPP) (*P* = 0.0389) elicited by surprised faces (Pearson's correlation coefficient).

No significant main effect of Emotion or interaction was seen for N170. The main effect of Site was significant (F (1, 21) = 12.51, *P* = 0.0019), suggesting that N170 is significantly more negative at T6 than at T5. We thus correlated N170 at T6 site (Figure 1) and IRI score. In general, a significant, negative correlation was apparent between IRI and N170. As seen in Table 2, IRI total scores correlated significantly and negatively with N170 elicited by angry (*P* = 0.0401) and surprised faces (*P* = 0.0026, Figure 2A). The Perspective taking scale correlated significantly and negatively with N170 elicited by happy (*P* = 0.0191) and surprised faces (*P* = 0.0166) (Table 2). Empathic concern scale correlated significantly and negatively with N170 elicited by happy (*P* = 0.0463), angry (*P* = 0.0016), surprised (*P* = 0.0002), and afraid faces (*P* = 0.0420) (Table 2).

**Table 2:** Correlations between empathy trait (Interpersonal Reactivity Index (IRI) score) and event-related potential (ERP) responses

**IRI score** **ERP responses**	**Total score**	**Subscale**			
		**Perspective taking**	**Fantasy**	**Empathic concern**	**Personal distress**
*N170*					
Happy faces	-0.30	-0.50^a^	0.00	-0.43^a^	0.01
Angry faces	-0.44^a^	-0.32	-0.16	-0.63^b^	-0.23
Surprised faces	-0.61^b^	-0.51^a^	-0.32	-0.71^b^	-0.26
Afraid faces	-0.33	-0.18	-0.29	-0.44^a^	-0.07
Sad faces	-0.32	-0.33	-0.14	-0.36	-0.11
*Early LPP*					
Happy faces	0.31	0.29	0.18	0.17	0.24
Angry faces	0.34	0.45^a^	0.12	0.17	0.21
Surprised faces	0.04	0.25	-0.10	-0.05	-0.01
Afraid faces	0.38	0.49^a^	0.21	0.26	0.10
Sad faces	0.27	0.41	-0.01	0.18	0.20
*Late LPP*					
Happy faces	0.49^a^	0.43^a^	0.39	0.46^a^	0.11
Angry faces	0.49^a^	0.27	0.48^a^	0.31	0.28
Surprised faces	0.44^a^	0.54^b^	0.23	0.31	0.16
Afraid faces	0.42	0.43^a^	0.21	0.39	0.17
Sad faces	0.44^a^	0.38	0.31	0.34	0.22

n = 22. ^a^Pearson correlation; *P* < 0.05. ^b^*P* < 0.01. LPP, late positive potential.

N170: T6 site; Early LPP: Pz site; Late LPP: Fz site.

### LPP

**Figure 3:**
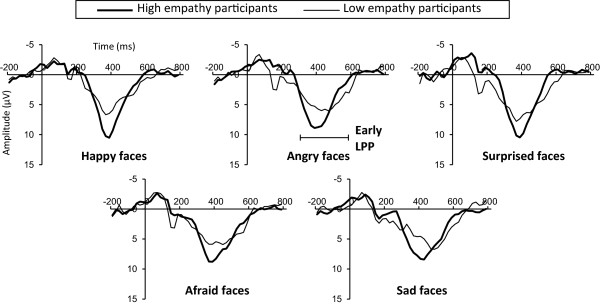
**Grand-averaged event-related potential (ERP) waveforms elicited at Pz.** High empathy participants (thick line, n = 12, range of total Interpersonal Reactivity Index (IRI) scores: 80 to 96) and low empathy participants (thin line, n = 10, range of total IRI scores: 54 to 78) were selected by total IRI score and labelled only for illustrative purposes.

For early LPP, no significant main effect of Emotion or interaction was evident. A main effect of Site was significant (F (1.59, 33.32) = 46.78, *P* = 0.0000), suggesting that early LPP is significantly more positive at Pz than at Fz (*P* = 0.0000), and Cz (*P* = 0.0380). We thus correlated early LPP at Pz (Figure 3) and IRI score. The Perspective taking scale showed significant, positive correlations with early LPP elicited by angry (*P* = 0.0373) and afraid faces (*P* = 0.0198) (Table 2).

**Figure 4:**
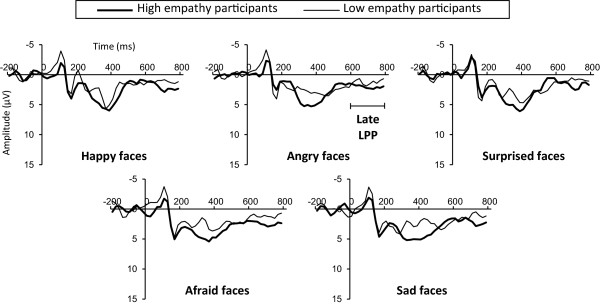
**Grand-averaged event-related potential (ERP) waveforms elicited at Fz.** High empathy participants (thick line, n = 12, range of total Interpersonal Reactivity Index (IRI) scores: 80 to 96) and low empathy participants (thin line, n = 10, range of total IRI scores: 54 to 78) were selected by total IRI score and labeled only for illustrative purposes.

For late LPP, no significant main effect of Emotion or interaction was seen. A main effect of Site was significant (F (1.36, 28.63) = 33.61, *P* = 0.0000), suggesting that late LPP is significantly more positive at Fz than at Cz (*P* = 0.0085) and Pz (*P* = 0.0000). We thus correlated late LPP at Fz (Figure 4) and IRI score. IRI total score correlated significantly and positively with late LPP elicited by happy (*P* = 0.0205), angry (*P* = 0.0221), surprised (*P* = 0.0389, Figure 2B), and sad faces (*P* = 0.0388) (Table 2). The Perspective taking scale correlated significantly and positively with late LPP elicited by happy (*P* = 0.0445), surprised (*P* = 0.0093), and afraid faces (*P* < 0.0448) (Table 2). The Fantasy scale correlated significantly and positively with late LPP elicited by angry faces (*P* = 0.0246) (Table 2). The Empathic concern scale correlated significantly and positively with late LPP elicited by happy faces (*P* = 0.0378) (Table 2).

### Behavioral responses

For response accuracies, a significant main effect was seen for Emotion (F (4, 84) = 4.68, *P* = 0.0019), showing that response accuracies were highest in response to surprised faces (mean = 99.34%, SD = 0.76%) and lowest in response to sad faces (mean = 98.20%, SD = 1.71%). Response accuracies did not show any significant correlation with IRI (all *P* > 0.05).

Reaction times also showed a significant main effect of Emotion (F (4, 84) = 11.67, *P* = 0.0000), appearing shortest in response to surprised faces (mean = 386.81 ms, SD = 52.23 ms) and longest in response to sad faces (mean = 440.30 ms, SD = 56.87 ms). Reaction times did not show a significant correlation with IRI (all *P* > 0.05).

Subjective rating showed a significant main effect of Emotion (valance: F (4, 84) = 76.81, *P* = 0.0000; arousal: F(2.92, 61.27) = 15.87, *P* = 0.0000). Valance rating showed that happy face was rated as the most pleasant expression, whereas angry face was rated as the most unpleasant expression (Table 3). Arousal rating revealed that happy face was rated as the most arousing face, whereas sad face was rated as the least arousing face (Table 3). No items of subjective rating showed any significant correlation with IRI (all *P* > 0.05).

**Table 3:** Subjective ratings

	**Valance**	**Arousal**
Happy faces	5.8 (2.7)	5.5 (1.9)
Angry faces	-5.0 (2.2)	5.2 (2.6)
Surprised faces	-0.1 (1.5)	4.2 (2.9)
Afraid faces	-3.2 (2.5)	3.1 (3.3)
Sad faces	-3.0 (2.7)	0.4 (3.2)

n = 22. Mean (SD).

## Discussion

The present study sought to clarify the relationship between empathy trait and attention responses to five facial expressions (happy, angry, surprised, afraid, and sad), by measuring N170 and LPP components as indices of attention.

### Empathy trait and N170

In the present study, clear N170 was elicited in response to all five facial expressions - happy, angry, surprised, afraid, and sad faces. In addition, N170 was more negative at the right posterior temporal area than at the left posterior temporal area. This is in line with previous findings [18,19,22] and supports the idea of Campanella *et al*. [19] that the perception of human faces is associated with the right posterior temporal area. However, N170 was not different depending on facial expressions in the present study. Some previous studies have reported that N170 is modulated by facial expression [34,35], while others have not found this association [36,37]. The present study supports the latter findings [36,37], suggesting that N170 is not different among facial expressions in the task of discriminating emotional facial expressions from emotionally neutral facial expressions.

Overall, N170 showed negative correlations with IRI for happy, angry, surprised, and afraid faces. The present finding thus suggests that individuals with high empathy trait pay attention more than those with low empathy from very early stage (around 170 ms after face onset), not only to angry face [6], but also to happy, surprised, and afraid faces. However, in response to sad faces, no significant correlation between IRI and N170 was seen. This might be because sad faces were the most difficult facial expression to discriminate from emotionally neutral faces in the present experiment, given that response accuracy was lowest and reaction time was longest in response to sad faces. In addition, sad faces were rated as the least arousing facial expression. More time might therefore be required for empathy trait to affect the attention processing of sad faces, as N170 reflects the very early stage of attention.

In terms of the relationship between each subscale of IRI and N170, the present study showed that N170 correlates with perspective taking and empathic concern scales, not with fantasy or personal distress scales. As mentioned earlier, the perspective taking scale represents attempts to take the perspectives of others, and thus reflects the cognitive aspect of empathy more than other subscales of IRI [16]. Interestingly, the perspective taking scale correlated with happy and surprised faces, but not with angry or afraid faces in the present study. Thus, the cognitive aspect of empathy might affect early processing of faces with positive (happy) or ambiguous (surprised) expressions, rather than with negative expressions (angry or afraid). Meanwhile, the empathic concern scale correlated with happy, angry, surprised, and afraid expressions, partly supporting the previous finding [6] of a negative correlation between empathic concern scale and N170 elicited by angry faces. Given that the empathic concern scale assesses the tendency to feel compassion for others [23], a willingness act altruistically might be strongly related to early processing of facial expressions, regardless of the valance of facial expressions.

### Empathy trait and LPP

Early LPP (300 to 600 ms) was greater at the medial parietal area than at the frontal and central areas. Typical early LPP was thus thought to be generated in the present experiment, as LPP is generally reported to be maximal at centro-parietal sites [9,13,15,38]. Meanwhile, late LPP (600 to 800 ms) was greater at the frontal area than at the central and parietal areas, inconsistent with the previous findings mentioned above [9,13,15,38]. Nonetheless, some ERP studies have reported frontal enhancement of LPP [39-41]. For example, Leutgeb *et al*. [40] suggested increased LPP at frontal sites relates to controlled attentional engagement. In addition, late LPP (>600 ms) seems to reflect elaborate processing of stimuli compared with early LPP (<600 ms) [14,15]. Taken together, frontal enhancement of late LPP is thought to reflect increased cognitive processing of stimuli. We thus suggest that the present oddball task to discriminate facial expressions from emotionally neutral faces as quickly as possible entailed cognitive and sophisticated attention.

In line with our hypothesis, the present study revealed generally positive correlations between IRI and LPP. In particular, late LPP correlated with IRI for all facial expressions presented in the present study - happy, angry, surprised, afraid, and sad faces. We thus suggest that individuals with high empathy pay attention more than those with low empathy in the late stage (600 to 800 ms after face onset) to surprised, afraid, and sad faces, as well as to happy and angry faces [5]. Given that frontal enhancement of late LPP mirrors cognitive processing as mentioned above, the present study also indicates that empathy trait affects cognitive and voluntary attention for processing of those five facial expressions. Meanwhile, early LPP correlated with IRI only for angry and afraid faces, unlike late LPP. Empathy trait seems to relate to obligatory attention only for negative and arousing facial expressions such as angry or afraid faces, as early LPP reflects obligatory capture of attention more than late LPP [14,15].

In addition, late LPP correlated with IRI for sad faces, while N170 did not. This finding supports our interpretation mentioned above, suggesting that the processing of sad faces takes longer than the time course of N170.

Investigating each subscale of IRI, the perspective taking scale showed greater correlations with LPP than other subscales of IRI. The fantasy scale correlated only with late LPP elicited by angry faces and the empathic concern scale correlated only with late LPP elicited by happy faces. Related to the correlation between the fantasy scale and LPP to angry faces, the present results are partly consistent with our previous study [5], which reported a correlation between the fantasy scale and late LPP elicited by discriminating angry and happy facial expressions. Attention to angry expressions in others is thus thought to be related to a tendency to be immersed in fiction. However, explaining why late LPP to only angry faces is related with fantasy scale is difficult, as is finding a supportive reason why late LPP to only happy faces correlated with empathic concern scale in the present study. Further research is warranted to explore which aspects of empathy are related to specific facial expressions.

### Empathy trait and surprised faces

Surprised expressions revealed stronger correlations between IRI and ERP responses (both N170 and LPP) than the other four facial expressions presented in the present study. We suggest that this might be because the valance of surprised faces is ambiguous. Previous studies [42-44] have reported that surprised faces can be interpreted as both positive and negative expressions, depending on context. For example, Neta *et al*. [44] reported that surprised faces are rated as more positively within the context of positive faces than within the context of angry faces. In the present study, surprised faces were presented only within emotionally neutral faces. The ambiguity of surprised faces might thus have been increased in the present study and individuals with high empathy might pay particular attention to surprised faces, in order to gauge the valance of the surprised face.

### Limitations and future directions

Several limitations must be considered when interpreting the results of the current study. First, the findings are correlational, so causal relationships remain undetermined. Second, a disgusted facial expression was not presented as stimuli in the current study, although this is one of the six basic facial expressions [27]. The relationship between empathy trait and the N170/LPP response elicited by disgusted faces thus remains unclear and future studies should address this question.

Although some neuroscience studies have reported individual differences in empathy [5-8], the current study is the first to investigated relationships between empathy trait and ERP response to various facial expressions. The high temporal resolution of ERP enabled us to clarify that empathy trait is related to the very early stage (reflected in N170) and late stage (reflected in LPP) of attention to facial expressions. Furthermore, since we measured empathy trait using IRI, the current study could address which facet of empathy relates to attention to facial expressions. As mentioned in Background, empathy has three facets: sharing of experience; mentalizing; and prosocial concern [30]. Prosocial concern has received less attention in neuroscience studies than the other two facets [30]. Interestingly, in the current study, N170 showed the strongest correlations with the empathic concern subscale among IRI subscales, reflecting the prosocial concern facet of empathy. The current result thus highlights the importance of the prosocial concern facet in empathy, suggesting that prosocial concern for others might affect the increase in attention to faces in very early stage, and *vice versa*.

## Conclusions

We found that IRI correlated negatively with N170 in response to happy, angry, surprised, and afraid faces, but correlated positively with LPP in response to happy, angry, surprised, afraid, and sad faces. This indicates that individuals with high empathy pay greater attention to various facial expressions than those with low empathy, from the very early stage (reflected in N170) to the late stage (reflected in LPP) of facial processing. In addition, the relationship between empathy trait and attention to face was strongest for the surprised facial expression, which might relate to the ambiguity of the surprised facial expression. Furthermore, N170 showed the strongest correlation with the empathic concern subscale among the IRI subscales, which is related to prosocial behaviour. We therefore suggest that among the facets of empathy, the prosocial concern facet in particular affects the increase in attention to facial expressions in the very early stage and *vice versa*.

## Abbreviations

ANOVA: analysis of variance; EEG: electroencephalography; EOG: electrooculography; EQ: The Empathy Quotient; ERP: event-related potential; fMRI: functional magnetic resonance imaging; IRI: Interpersonal Reactivity Index; LPP: late positive potential; VAS: Visual Analog Scale.

## Competing interests

The authors declare that they have no competing interests.

## Authors’ contributions

DC and SW contributed to the design of the experiment. DC performed the experiments, analyzed the data and wrote the manuscript with advice from SW. TN, MM, and YE participated in the discussion and preparation of the manuscript. RM helped coordinate research activities. All authors read and approved the final manuscript.
